# Study on Short Fatigue Crack Behaviour of LZ50 Steel Under Non-Proportional Loading

**DOI:** 10.3390/ma13020294

**Published:** 2020-01-08

**Authors:** Bing Yang, Zhen Liao, Shoune Xiao, Guangwu Yang, Tao Zhu, Xiangning Zhang

**Affiliations:** 1 State Key Laboratory of Traction Power, Southwest Jiaotong University, Chengdu 610031, China; liaozhen6@163.com (Z.L.); gwyang@swjtu.edu.cn (G.Y.); zhutao034@swjtu.edu.cn (T.Z.); 2 CRRC Tangshan Co. Ltd., Tangshan 063000, China; sjc-zhangxiangning@tangche.com

**Keywords:** LZ50 steel, short cracks, replica test, non-proportional loading

## Abstract

The low cycle fatigue tests using the replica technique for LZ50 steel under non-proportional cyclic loading were carried out, and eight groups of effective test data were obtained. The evolution behaviour of short cracks was studied based on the effective short cracks criterion. The results show that short cracks generally originate in the grain or along the grain boundary. At the microstructural short crack stage, the crack propagation is influenced strongly by the microstructure of the material, and the growth rate of the short crack slows down several times according to the number of obstacles encountered. At the physical short crack stage, the crack propagation breaks through the banded structure of pearlite. Thus, the dominant effective short fatigue crack is formed, and the crack growth rate increases rapidly. Based on the modified parameters of the uniaxial short crack model, an approach is presented to calculate the growth rate of short cracks under multi-axial non-proportional loadings, and the new model can consider the non-proportional factor *F*. The fitting results of the multi-axial microstructural obstacles model are compared with test data. The comparison results show that this model can reflect the trend of short fatigue crack propagation rate under non-proportional loadings.

## 1. Introduction

Multi-axial fatigue is one of the main factors that causes destruction of the metallic structure in service. Even if some components are under uniaxial loading, the local areas of these components are still in a state of multi-axial stress due to the complex geometry of the components. The alternating action of multi-axial stress is the main cause for fatigue failure of the components. Meanwhile, fatigue failure is a developing process, mainly including the initiation and propagation of short cracks, the instability propagation of long cracks, and the final fracture. The initiation and propagation of short cracks account for the majority of fatigue life [1]. However, the long crack belongs to the rapid growth stage, which has little influence on fatigue life. Therefore, the analysis of short cracks’ propagation behaviour has important engineering value.

Gough and Pollard [2] first systematically studied multi-axial fatigue. Then, some scholars [3,4,5] carried out the research of multi-axial fatigue and mainly focused on the S-N curve of multi-axial fatigue under proportional loading. With the famous Manson-Coffin law [6,7] proposed, the equivalent strain parameter was taken as a parameter of multi-axial damage, and the fatigue life under multi-axial loadings was estimated by using Mason-Coffin formula. Although it was indeed able to obtain good results from the multi-axial life under proportional loading according to these methods, the fatigue life under non-proportional loading could not get satisfactory results by utilizing these methods. As proposed by Brown and Miller [8], a critical plane could be used to predict multi-axial low cycle fatigue life, and the maximum shear strain parameter and normal strain parameter causing the crack initiation and propagation of materials were considered in the method. In this case, this method was widely used to obtain many fatigue life prediction models under non-proportional loading [9,10,11,12]. However, these models did not consider the effect of microstructure obstacles on short fatigue cracks propagation.

In the present study, taking LZ50 steel, a commonly used material for railway axles in China, as an example, the low cycle fatigue test was carried out under axial-torsion loading to study the short fatigue cracks’ behaviour of the material. Moreover, a new approach considering the microstructure obstacles of the material is put forward to calculate short fatigue cracks’ growth rate under non-proportional loadings.

## 2. Experimental Details 

### 2.1. Materials and Specimens

**Figure 1:**
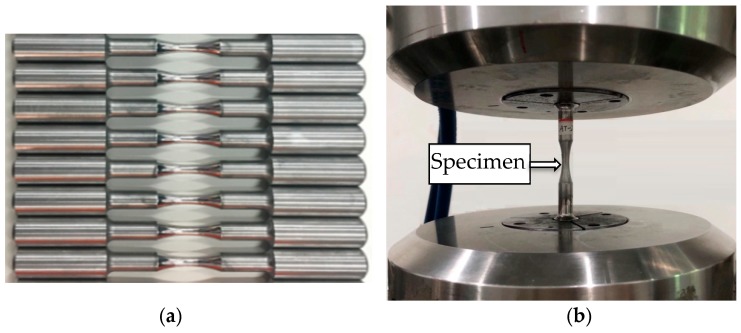
(**a**) Specimens and (**b**) a specimen clamped on the test machine.

The LZ50 steel used in the railway axle industry is a carbon structural material, with the following chemical compositions (wt.%): C 0.47, Si 0.26, Mn 0.78, Cr 0.02, Ni 0.028, Cu 0.15, Al 0.021, P 0.014, S 0.007. Its material properties are shown in Table 1. Referring to the previous studies [13,14,15], the material presents the typical ferrite and pearlite structure and the rich pearlite banded structure distributed along the rolling direction. The specimens employed in this study were machined to a smooth funnel shape with a minimum diameter of 6 mm, and the radius of curvature is 50 mm according to ASTM standard E466-15 [16], which were shown in Figure 1.

### 2.2. Experimental Details

**Figure 2:**
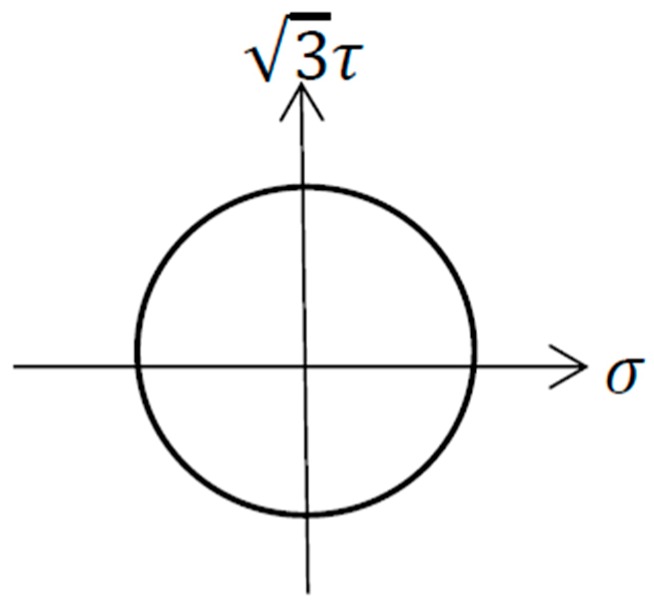
Axial-torsion loading path.

All fatigue tests were carried out on a CMT 5105 universal testing machine (MTS). The fatigue tests were performed in the mode of load control at room temperature. The Sinusoidal wave with a frequency of 2 Hz was used for cyclic loading. One axial-torsion non-proportional loading path was set in the fatigue tests, as shown in Figure 2.

The stress-strain curve was obtained from the fatigue tests. The corresponding theoretical calculation formulas are listed as follows.

Axial stress σ and shear stress τ conversion:

(1) $$\sigma =\frac{4F}{\pi d^{2}}$$

(2) $$\tau =\frac{16T}{\pi d^{3}}$$

where, *F* and *T* are the applied axial load and the torque, respectively. *d* is the diameter of the specimen.

Axial strain ε and shear strain γ conversion:

(3) $$\varepsilon =\frac{l-l_{0}}{l_{0}}$$

(4) $$\gamma =\frac{d\alpha }{4l_{0}}$$

where, *l_o_* and *l* are the total lengths of the specimen before and after deformation, respectively. *α* is the torsional angle.

The Von Mises equivalent stress, $\sigma_{eq}$, was used to multi-axial fatigue stress analysis, and it can be expressed as:

(5) $$\sigma_{eq}=\sqrt{\sigma^{2}+3\tau^{2}}$$

According to the above equations, the relevant parameters were calculated as shown in Table 2. The maximum equivalent stress $\sigma_{eq}=480$ MPa, which is greater than flow stress. Therefore, axial-torsion tests used in the current study are relying on low cycle fatigue criteria.

**Figure 3:**
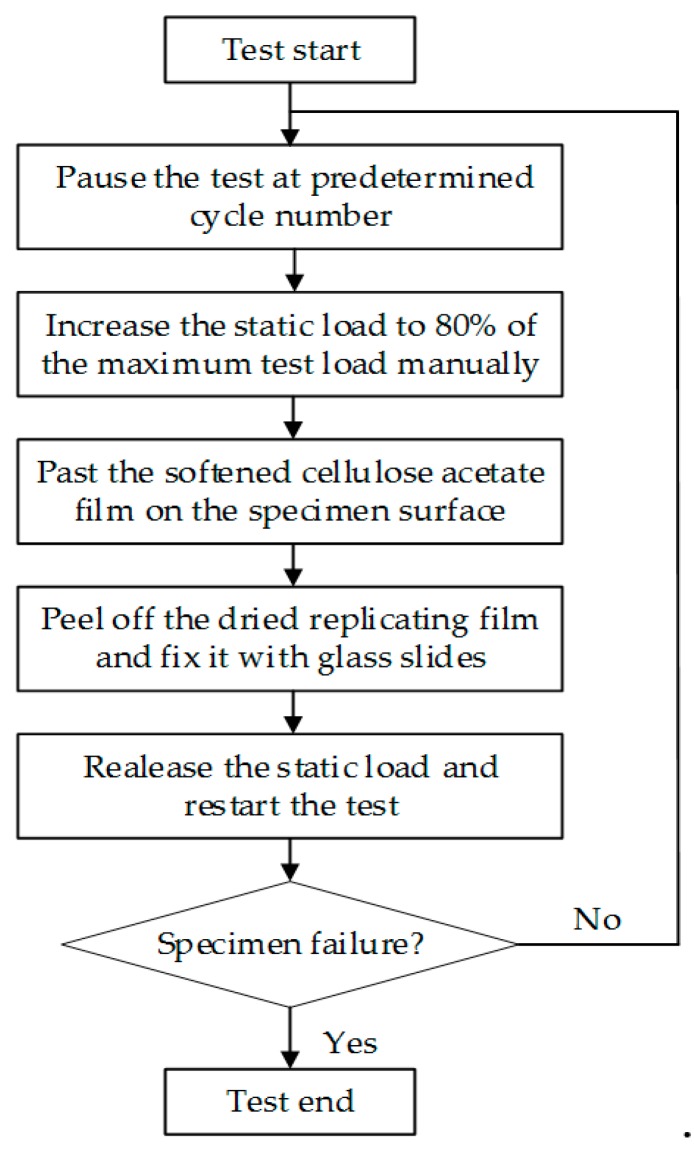
The operation steps of the replica test.

The fatigue replica tests were performed under non-proportional cyclic loading to study short crack growth. The tests were paused at a predetermined cycle number to make a replica of the specimen surface. To facilitate the replicating process, a static tensile load of 80% of the maximum test load was manually applied to ensure that the cracks were fully open [17]. A cellulose acetate film softened by acetone was pasted on the surface of the specimens to record the initiation and propagation of the cracks. The replica films could only be removed from the specimen surface after they are completely dry. A confocal laser scanning microscope OLYMPUS LEXT OLS4100 (Tokyo, Japan) was used to observe the replicas. Through backtracking the replica films, the crack morphologies, crack lengths and crack initiation sites were determined. The interval for replicating could be determined with reference to the fatigue life of a specimen under the continuous fatigue test load. For every replica specimen, more than 12 effective replicas were obtained to analyze the initiation and propagation of the short cracks. The replica tests were terminated until the specimens were completely fractured. The detailed step of the replica test is shown in Figure 3.

## 3. Results and Discussion

### 3.1. Experimental Data

**Figure 4:**
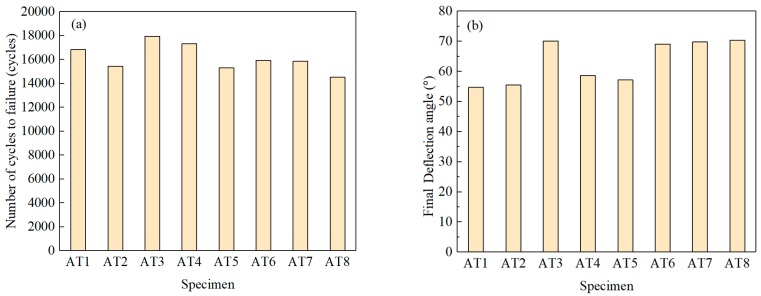
Test results of low-cycle fatigue under axial-torsion loading: (**a**) Fatigue life and (**b**) Final deflection angle (AT stands for axial-torsional test).

**Figure 5:**
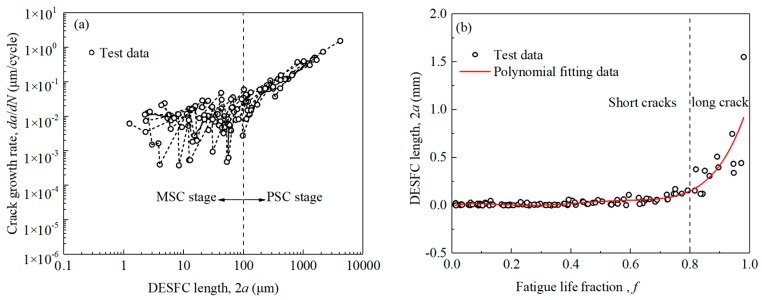
The relationship between the dominant effective short fatigue crack (DESFC) length and (**a**) the crack growth rate, and (**b**) the fatigue life fraction, which is the ratio of cycles number to fatigue life.

The fatigue life, crack length, deflection angle and other related data were obtained according to the replica test results, and the total fatigue life and final (permanent) deflection angle of each specimen are shown in Figure 4. To analyze the law of short cracks’ growth, eight sets of axial-torsion non-proportional test data are plotted in Figure 5. The crack length 2*a* is regarded as the total surface crack length, and the deflection angle α refers to the angle which the axis of the specimen rotates counterclockwise to the line, connecting both ends of the crack tips.

As shown in Figure 4a, the number of failure cycles for specimen was less than 10^5^, so the tests are subject to low cycle fatigue failure. As shown in Figure 4b, the range of the angle between fracture direction and specimen axis was from 55°–70°, which results from the combined influence of the cyclic tension-compression load and the cyclic torsion load. It can be seen from Figure 5a that at the microstructural short crack (MSC) stage, the crack growth rate slows down periodically when the corresponding crack lengths are close to the sizes of ferrite grain or to the distance between two rich pearlite bands. At the physical short crack (PSC) stage, the crack growth rate increases without slowing down. As can be seen from Figure 5b that the initiation and propagation of short crack growth account for about 80% of the fatigue life, which confirms the significance of studying the behaviour of the short cracks.

### 3.2. Fracture Observation

**Figure 6:**
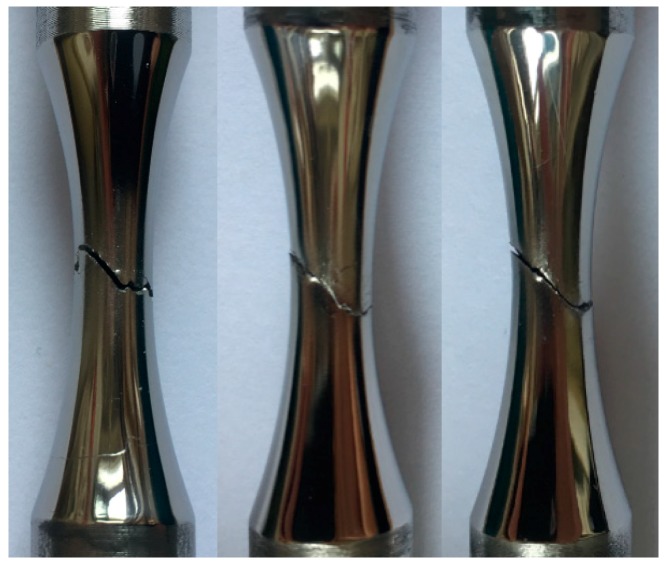
Fractography after the fatigue test.

In the fatigue tests under combined loading of axial and torsion, the angles between the fracture surface and the main axis for the specimens are close to one another, and the macroscopic surface morphology of some specimens was shown in Figure 6. It can be observed from the graph that there is a certain deflection angle between the fracture plane and the axial direction of the specimen, which was around 60°. Compared with the static load failure, the specimen shows no obvious plastic deformation, and the two fracture surfaces can match each other well.

**Figure 7:**
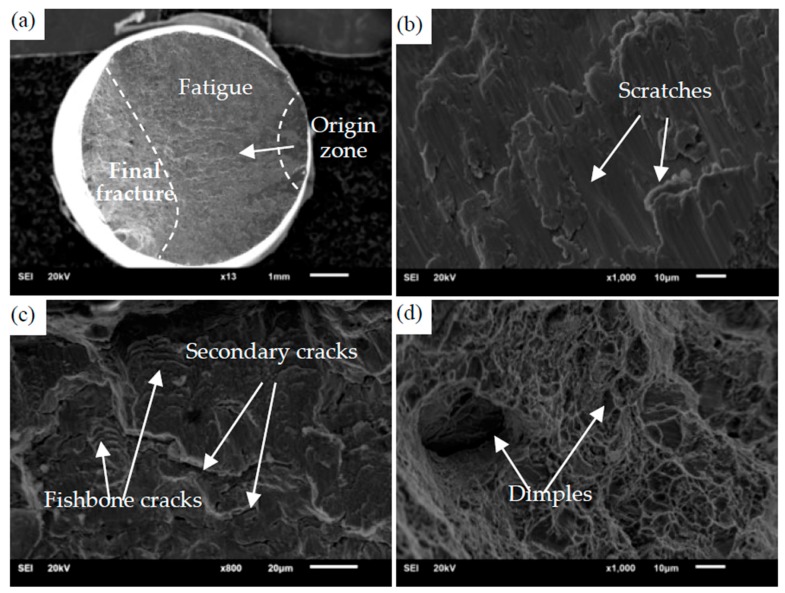
SEM images of the fracture. (**a**) Overall fracture morphology; (**b**) the morphology of crack origin zone and extension zone; (**c**) the typical morphology of crack propagation zone; (**d**) the typical morphology of transient fracture zone.

To further analyze the fatigue fracture behaviour in the depth direction, the scanning electron microscope was used to observe the fracture. Firstly, Figure 7a shows that fatigue fracture is composed of origin zone, extension zone and transient fracture zone, and the form of failure is single crack source failure. In the origin zone, there are obvious grayish-black characteristics and a flat fracture due to repeated tension-compression and reversed torsion during loading. Secondly, some useful information can be observed in Figure 7b,c; that is the fatigue crack growth zone presented typical morphologies, such as scratches, fishbone cracks. At the same time, there are many secondary cracks in the extension zone. Lastly, it can be seen from Figure 7d that in the transient fracture zone there is a typical dimple shape, which is consistent with the characteristics of the fatigue instantaneous fracture zone under uniaxial tension and compression. This indicates that the cyclic action of the normal stress causes more of the fracture of the specimen in the later stage of crack formation than the shear stress.

### 3.3. Initiation and Propagation of Short Fatigue Cracks

This research focuses on microstructural and physical short cracks, which have a great influence on fatigue life. Microstructural short cracks are those with length scales comparable to microstructural characteristic sizes, such as grain size. Physical short cracks are sufficiently long about the microstructural short cracks but are small enough that their growth rate differs from those of long cracks [1,18]. Based on the principle of short fatigue cracks proposed by Zhao [19,20], the results of replica tests were analyzed. The test results revealed that the initiation of the effective short fatigue cracks (ESFC), the formation and propagation of dominant ESFC (DESFC), which makes a direct contribution to fatigue failure.

**Figure 8:**
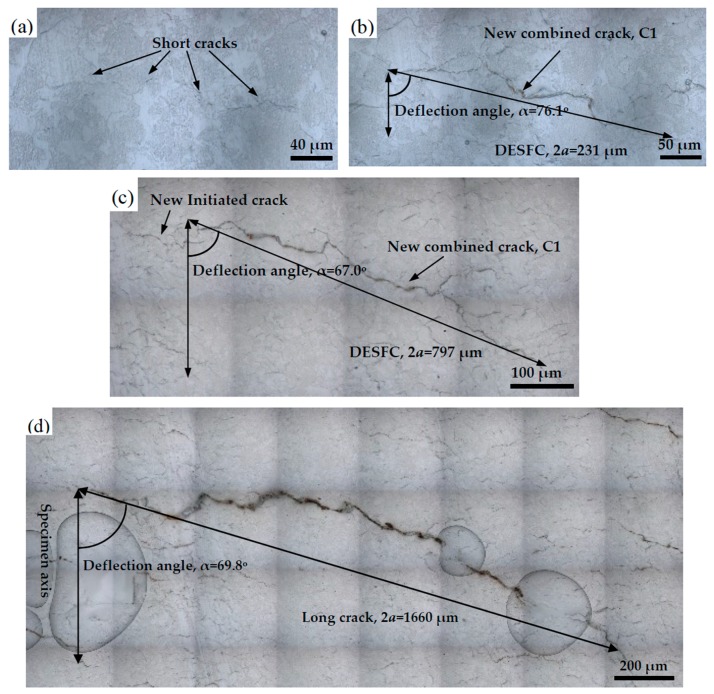
Images of fatigue crack under different cycles: (**a**) N = 3000 cycles, (**b**) N = 115,000 cycles, (**c**) N = 13,000 cycles, and (**d**) N = 15,000 cycles.

Figure 8 shows a representative initiation and propagation process of short cracks taking specimen AT 7 as an example. Figure 8a shows the replica image in the MSC stage, while Figure 8b,c shows those in the PSC stage. Figure 8d shows the replica image of a long crack. It can be seen that in the MSC stage, the crack propagated in a transgranular mode and the crack growth path exhibited a zig-zag shape. The microstructure of grain boundary and banded structure acted as the main obstacles to crack propagation in this stage. In Figure 8b, the crack tip of ESFC passed through the banded structure, and the ESFCs connected with one another and transformed into one crack (C1). The DESFC (C1) has been established, and the length and deflection angle of C1 were 231 μm and 76.1°, respectively. As shown in Figure 8c, with the increase of cycles, the DESFC entered the rapid growth stage, and the loading condition has become the main factor affecting its propagation. The DESFC length and deflection angle were 797 μm and 67°, respectively, at this moment. The last replica image before fracture was shown in Figure 8d. It can be seen that the crack length and deflection angle were 1660 μm and 69.8°, and it was already in the long crack stage. Then, the long crack propagated forward rapidly until the specimen fractured.

## 4. The Short Cracks Growth Model Under Uniaxial Loading

### 4.1. Basic Formula of Growth Rate

The propagation process of short fatigue cracks has strong dispersion and uncertainty due to the influence of many factors. In particular, in the MSC stage, the crack growth path exhibits a zig-zag shape, which does not meet the linear elastic fracture mechanics. In the PSC stage, the linear elastic fracture mechanics cannot describe the crack propagation due to the influence of the plastic zone at the crack tips. Therefore, the traditional linear elastic fracture mechanics cannot accurately describe the propagation law of short fatigue cracks.

Due to the failure of linear elastic mechanics, *J* integral was selected as the driving force of crack propagation in the region near the crack tip on the critical surface. The conservation principle of the J integral could be easily converted to the far field for calculation.

It is assumed that the crack growth rate in a cyclic period [21] is:

(6) $$\frac{da}{dN}=CJ^{r}$$

where, *C* is material constant.

At the critical plane, the material should be in the stage of elastoplastic deformation. Therefore, the relationship between *J* integral and the total strain energy density *W_t_* in the far field can be expressed as:

(7) $$J=2\pi Y^{2}W_{t}a$$

where, *Y* is geometric parameter for crack. *a* is surface crack length.

After a cycle, the crack growth rate is:

(8) $$\frac{da}{dN}=C{\left(2\pi Y^{2}W_{t}a\right)}^{r}$$

Set *A* = *C*(2*πY*^2^), then Equation (6) can be converted into as:

(9) $$\frac{da}{dN}=C{\left(W_{t}a\right)}^{r}$$

where, *r* is the total strain energy utilization index, and *C* is the material-related coefficient. Therefore, Equation (9) is the basic formula of fatigue short crack growth rate under uniaxial loading.

### 4.2. The Formula of Short Fatigue Crack Growth Rate

The fatigue behaviour of short cracks is not similar to that of long cracks in linear elastic mechanics. Cyclic *J*-integral was selected as the driving force of crack propagation in the local area near the tip of the critical plane for this reason. The conservation principle of *J*-integral can be easily converted to the far-field for numerical calculation. Based on the principle of effective short fatigue cracks, Zhao [19,20] proposed a growth rate model of short fatigue cracks in MSC stage and PSC stage, considering the deceleration effect on crack growth in MSC stage and the synthesis of the effective short crack in PSC stage.

On the basis of Zhao’s research results, Yang [22] proposed a short crack growth rate model that included the microstructure barriers *d*_i_ and the damping effect function *f*_i_(△*d*_i_):

(10) $$\frac{da}{dN}=G_{0}+A{\left[\Delta W_{t}a-\Delta W_{t}\overset{n}{\underset{i=1}{\sum }}f_{i}\left(\Delta d_{i}\right)d_{i}\right]}^{m}$$

where, *G*_0_ is the basic growth rate in the first cycle at the microstructural barriers scale, △*W*_t_ is the far-field total cyclic strain energy density, i is the serial number of microstructural barriers, *n* is the total number of microstructural barriers, *A* and *m* are constants relating to materials.

The damping function in Equation (10) can be expressed as:

(11) $$f_{i}\left(\Delta d_{i}\right)=1-\left(\frac{d_{i}-\Delta d_{i}}{d_{i}}\right)a_{i}$$

where, *d*_i_ is the characteristic size of the ith microstructural barrier, △*d*_i_ is the size of DESFC tip beyond the previous microstructural barriers, *α*_i_ is a constant value relating to microstructural barriers and test data. Equation (11) can reflect the characteristic that short cracks are arrested by microstructural barriers.

**Figure 9:**
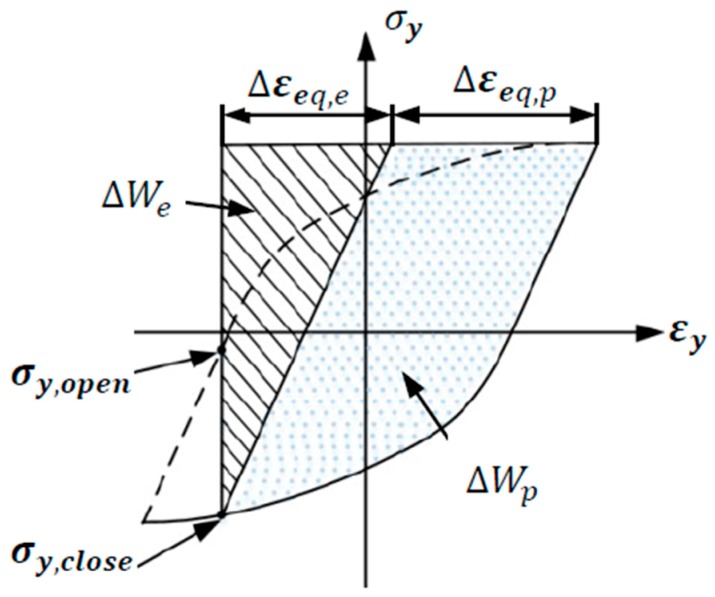
Effective elastic and plastic energy density [15].

In the calculation of the total deformation energy density function in the far field, crack growth is caused by crack opening when the equivalent stress is positive, and crack closure has no contribution to crack growth when the equivalent stress is negative. In a single cycle, the positive area of the equivalent stress-strain hysteresis loop and the coordinate axis is equal to the value of the total strain energy density in the far field, as shown in Figure 9. Under symmetrical cyclic loading, the total strain energy density can be expressed as:

(12) $$W_{t}=\frac{E}{8}{\left(\Delta \varepsilon_{eq,e}\right)}^{2}+K_{n}^{\prime }\frac{{\left(\Delta \varepsilon_{eq,p}\right)}^{n^{\prime }+1}}{n^{\prime }+1}$$

where *E* is Young’s modulus, $K_{n}^{\prime }$ is uniaxial cyclic strengthening coefficient, $n^{\prime }$ is uniaxial cyclic hardening index, $\Delta \varepsilon_{eq,e}$ and $\Delta \varepsilon_{eq,p}$ are the elastic equivalent strain amplitude and the plastic equivalent strain amplitude, respectively. For LZ50 steel, the values of $K_{n}^{\prime }$ and $n^{\prime }$ are 1117.54 MPa and 0.225, respectively [23]. The total equivalent strain amplitude can be expressed as:

(13) $$\Delta \varepsilon_{eq}=\Delta \varepsilon_{eq,e}+\Delta \varepsilon_{eq,p}$$

## 5. The Short Cracks Growth Model Under Multi-Axial Loading

### 5.1. Modification of Relevant Mechanical Parameters

There is a phase difference between the axial strain and the rotational strain under the multi-axial non-proportional loadings, which makes the main axis of the strain in the critical plane change continuously during the rotation process. As a result, the shear strain and the normal strain on the critical plane cannot reach the maximum value at the same time. According to the previous research experience [24], the modified uniaxial loading test parameters can be directly applied to multi-axial non-proportional loading. The feasibility of this method has been confirmed by relevant literature [25,26,27,28]. The modified non-proportional cyclic strength coefficient can be expressed as:

(14) $$K_{n}=\left(1+gF\right)K_{n}^{\prime }$$

where $K_{n}^{\prime }$ is the cyclic strain hardening coefficient for uniaxial loading, and *g* is the crossing hardening coefficient, measured according to the multi-axial loading. 

The parameter *F* is the rotation factor [24] defined as the ratio of the shear strain range on a plane bisecting to the maximum shear strain range:

(15) $$F={\left\{\frac{\lambda^{2}+{\left(1+\mu \right)}^{2}-\sqrt{{\left[{\left(1+\mu \right)}^{2}-\lambda^{2}\right]}^{2}+{\left[2\lambda \left(1+\mu \right)cos\emptyset \right]}^{2}}}{\lambda^{2}+{\left(1+\mu \right)}^{2}+\sqrt{{\left[{\left(1+\mu \right)}^{2}-\lambda^{2}\right]}^{2}+{\left[2\lambda \left(1+\mu \right)cos\emptyset \right]}^{2}}}\right\}}^{2}$$

where *λ* is the ratio of shear strain amplitude to normal strain amplitude, *μ* is Poisson’ ratio, and *Φ* is a phase difference between shear strain and normal strain.

Based on the above derivations, the far-field strain energy density function is modified using Equation (12). The short fatigue crack growth rate under multi-axial loading can be obtained using Equation (10).

### 5.2. Experimental Verification of the Multiaxial Short Cracks Growth Model

Short cracks’ propagation is mainly affected by the ferrite grain boundary and the rich pearlite banded structure. To simplify the description, subscript 1 and 2 are used to represent these two kinds of microstructure barriers, respectively. The model under axial-torsion loading can be expressed as follows:

(16) $$\frac{da}{dN}=G_{01}+A_{1}\Delta W_{t}{\left[a-f_{1}\left(\Delta d_{1}\right)d_{1}\right]}^{m_{1}}$$

(17) $$\frac{da}{dN}=G_{02}+A_{2}\Delta W_{t}{\left[a-f_{2}\left(\Delta d_{2}\right)d_{2}\right]}^{m_{2}}$$

To solve the unknown parameters, *G*_0_ can be moved to the left side of the equation, and then take the logarithm of both sides, Equations (16) and (17) can be converted into the following:

(18) $$log\;log\;\left(\frac{da}{dN}-G_{01}\right)\;=log\;log\;\left(A_{1}\Delta W_{t1}\right)\;+m_{1}\;log\;log\;\left(a-f_{1}\left(\Delta d_{1}\right)d_{1}\right)\;$$

(19) $$log\;log\;\left(\frac{da}{dN}-G_{02}\right)\;=log\;log\;(A_{2}\Delta W_{t2})\;+m_{2}\;log\;log\;\left(a-f_{2}\left(\Delta d_{2}\right)d_{2}\right)\;$$

**Figure 10:**
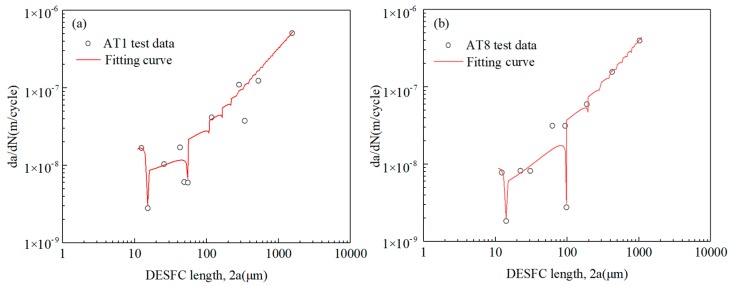
Comparison of the test data and fitting curve using the short cracks model with the non-proportional factor: (**a**) specimen AT1 and (**b**) specimen AT8.

Since d*a*/d*N*, *a*, *d*_1_, *d*_2_, can be determined by using replicating test data, and Δ*W*_t1_, Δ*W*_t2_ can be calculated by solving Equation (12), then by combining the exhaustive method and the least square method, the relevant parameters of Equations (18) and (19) can be obtained. Take 2 sets of test data as examples to calculate the unknown parameters as shown in Table 3, and compare the fitting curve of the fitted short cracks’ growth rate with the test data as shown in Figure 10. Microstructural barriers model under multi-axial loading can describe the periodic deceleration behaviour of growth rate in both the MSC stage and the PSC stage.

## 6. Conclusions

(1) Based on the replica test results using LZ50 steel specimens under non-proportional loadings, it can be seen that most parts of the fatigue life were consumed by the initiation and propagation of short cracks. In the MSC stage, the growth rate of DESFC slowed down periodically, and the crack length was consistent with the microstructural characteristic sizes of the material. In the PSC stage, the growth rate of short cracks increased with driving force without showing the influence of the microstructure obstacles.
(2) By modifying the mechanical parameters in the uniaxial short crack growth rate model, the model of short crack growth rate under multi-axial non-proportional loadings was obtained. The fitting growth rate curve of the multi-axial short crack reflects the periodic deceleration characteristics of the DESFC, which is in good agreement with the test data.

## Figures and Tables

**Table 1:** The material properties of LZ50 steel.

Poisson Ratio (-)	Young’s Modulus (GPa)	Flow Stress (MPa)	Ultimate Tensile Stress (MPa)
0.31	210	330	629

**Table 2:** Test conditions of low-cycle fatigue under axial-torsion loading.

Specimens	*P*_max_ (N)	$\;\sigma_{a}\;(\mathrm{MPa})$	$\;R_{\varepsilon }\;$	*T*_max_ (N⋅m)	$\;\sqrt{3}\tau_{a}\;(\mathrm{MPa})$	$\;R_{\gamma }\;$	$\;\sigma_{eq}\;(\mathrm{MPa})$	Phase Angle (^o^)
AT1~AT8	9600	340	−1	8.3	340	−1	480	90

**Table 3:** Parameters of the multi-axial short fatigue growth rate model for AT1 and AT8.

Specimen	AT1	AT8
***d*_1_ (μm)**	15.20	14.09
***G*_01_ (μm/cycle)**	2.80 × 10^−^^3^	1.84 × 10^−^^3^
***A*_1_**	2.38	1.94
$\;\Delta W_{t1}$ **(MPa·μm)**	3.2 × 10^−^^2^	3.09 × 10^-2^
***m*_1_**	2.13	2.33
***α*_1_**	0.35	0.38
***d*_2_ (μm)**	54.8	97.51
***G*_02_ (μm/cycle)**	6 × 10^−^^3^	2.77 × 10^−^^3^
***A*_2_**	1.02	1.36
$\;\Delta W_{t2}$ **(MPa·μm)**	3.18 × 10^−^^3^	2.83 × 10^−^^3^
***m*_2_**	1.05	1.06
***α*_2_**	0.33	0.27
